# The Phenomenology of Group Stalking (‘Gang-Stalking’): A Content Analysis of Subjective Experiences

**DOI:** 10.3390/ijerph17072506

**Published:** 2020-04-06

**Authors:** Lorraine Sheridan, David V. James, Jayden Roth

**Affiliations:** 1 School of Psychology, Curtin University, Perth, WA 6102, Australia; jayden.roth@student.curtin.edu.au; 2 Theseus Risk, Cavalier Court, Cheltenham SN14 6LH, UK; drdvjames@gmail.com

**Keywords:** phenomenology, stalking, gang-stalking, group stalking, prevalence, psychological sequelae

## Abstract

Epidemiological data suggest that as many as 0.66% of adult women and 0.17% of adult men in the western world may suffer the subjective experience of being group-stalked (‘gang stalked’) at some point in their lives. Yet the gang stalking experience has been subject to little scientific study. This paper reports an attempt to elicit the core phenomena involved in gang-stalking by allowing them to emerge de novo through the qualitative analysis of accounts of individuals who describe being gang-stalked. Fifty descriptions of gang-stalking that satisfied study inclusion criteria were identified from the internet and subjected to content analysis. Twenty-four core phenomena were elicited, together with 11 principal sequelae of the experience of being gang-stalked. These were then divided into groups, producing a framework for the phenomena of the gang-stalking experience. The results were compared with frequencies of the same categories of experience then extracted from the original data of the only previous study on gang-stalking phenomena. Whilst the methodology of the current study was more rigorous, the core phenomena were similar in each. The current study confirmed the seriousness of the sequelae of the gang-stalking experience. These support the need for further exploration of the phenomenon, for which this study forms a basis.

## 1. Introduction

Stalking denotes a pattern of repeated, unwanted intrusion by one person into the life of another in a manner that causes distress, disruption, or fear [1,2]. The concept of stalking was introduced in the late 1980s to describe a form of interpersonal aggression that, although common through the ages, had come to be socially unacceptable in the western world after the recognition of equal rights for women and the prosecution of domestic violence. To that extent, stalking is a social construct that arose in a particular social and cultural context [3].

Since the turn of the millennium, another term linked to stalking has gained currency in the media and on the world wide web—that of ‘group’ or ‘gang’ stalking. Stalking generally involves a single stalker who may occasionally recruit others into stalking by proxy, their involvement usually being unwitting [4]. By contrast, reports of group or gang-stalking describe stalking by multiple individuals who engage in a shared endeavour with a group purpose. For research purposes, the number is taken as three or more, although in many instances those suffering from the phenomenon have reported the involvement of far greater numbers [5].

Being stalked by individuals is a relatively common experience in the western world. The prevalence in different studies varies according to the definition used, the methodology employed, and the population or sub-population sampled. But even the most conservative estimates suggest that 8% of women and 2% of men are stalked at some point in their lives [6,7], and other studies from a range of western countries have found rates that were twice as high [8,9,10,11,12,13,14,15]. By contrast, indications as to the prevalence of gang-stalking experiences are few in number. In an anonymous questionnaire that was completed online by 1040 self-defined adult victims of stalking [5], 12.3% of respondents reported group or gang-stalking. A US Department of Justice prevalence study, which used a tight definition of stalking that required the victim to experience fear, found that 6.8% reported stalking by three or more people, and were “unable to identify a single offender” or “could not identify an offender who was singularly responsible” [16]. The US study also examined cases where the behavioural part of their definition of stalking was satisfied, but not the fear component. When these cases were added to those defined as stalking, the total figure for those reporting an inability to identify a single offender or offender who was singularly responsible was 12.5% [17], which is similar to that found in the questionnaire study. The gang-stalking phenomenon would thus appear to be relatively common. Yet, we could find only one empirical study of group or gang-stalking in the published literature [5]. By contrast, a Google search for “gang-stalking” conducted on 5 February 2020 produced 7,550,000 ‘hits’.

Stalking by individuals has been found to result in high rates both of psychological distress and lasting psychiatric morbidity, in particular post-traumatic symptomatology and depression [1,4,10,18,19,20]. The one study to examine the psychological sequelae of the experience of being gang stalked found that individuals who had been group or gang stalked scored significantly higher on ratings of depressive symptoms, post-traumatic symptomatology and adverse impact on social and occupational functioning than those who were individually stalked [5]. The only other published study of gang-stalking samples that we could locate detailed four cases reported by the media of men who had engaged in extreme violence as a response to the perception that they were the target of gang stalkers [21]. Both Sheridan and James [5] and Sarteschi [21] concluded that the subjects of their studies were suffering or had suffered from severe psychological distress in the context of their subjective gang-stalking experiences.

Despite the experience of gang-stalking being so widely reported and the evidence of its harmful effects on individuals and on society at large, there is a dearth of research into its nature and into the components that constitute the experience. Sheridan and James’ study [5] appears to be the only one to have investigated this central feature of this subject. As with most studies of victim experiences, their study was based upon questionnaire data from self-selected respondents. Whilst the questionnaire included free-text items for describing subjective experiences, its bulk text comprised a collection of information on specific pre-defined data points. Those concerning the types of intrusive behaviour and experiences that constitute stalking were taken from Spitzberg’s [22] meta-analysis, which extracted data for this part of his project from 43 studies. The focus of both the separate studies and of the meta-analysis was on the phenomenon of being stalked by a single individual. As such, Sheridan and James’ study in effect imposed a classification of phenomena on the questionnaire respondents and, as such, may potentially have excluded experiences amongst gang-stalked respondents that did not fit into the standard pattern reported by those stalked by individuals.

In order to overcome this problem, we decided to conduct a study of the experiences of those reporting gang-stalking that would allow the components of their experience to emerge de novo from their detailed accounts, rather being shoe-horned into a preconceived framework. This follows the phenomenological approach expounded by Mullen [23] in which subjective phenomena are approached through a descriptive phenomenology, free of the restraints of existing classification systems. The main aim of the study was to identify the phenomena that constitute the subjective experience of gang-stalking, free from assumptions based on the cases of those stalked by individuals. A second aim was to allow the emergence of the psychological and behavioural sequelae of gang stalking deemed most worthy of reporting by those describing their gang-stalking experiences, and which therefore were likely to be the most concerning to them. A subsidiary aim was to establish whether the experiences of the group selected for this study differed from those of the sample acquired by Sheridan and James [5] from a questionnaire survey, or whether the two were part of the same phenomenon.

## 2. Method

The study involved a mixed qualitative and quantitative design using content analysis with a sample of self-published accounts of gang-stalking extracted from the Internet. This research was approved by the Human Research Ethics Committee of Curtin University (RDHS-79-16).

### 2.1. Selection of Sample

A sample of 50 written accounts was considered sufficient to capture the overall essence of participant experiences while also giving the data to have adequate quantifiable merit [24]. Self-published accounts of gang-stalking were selected from within websites and web forums on the internet via purposive convenience sampling using Google searches of websites, blogs, and forums with the search terms “gang-stalking”, “group stalking”, “gang-stalking stories”, “victims of gang-stalking”, “targeted individual”, “TI”, and various combinations thereof (“targeted individual”, or TI, is a term by which some people reporting gang-stalking refer to themselves). More than 20 million hits were revealed in the initial search. Fifty personal accounts of gang-stalking were identified for use in the study by following the first 250 relevant links. All links were investigated, whatever their type (personal webpages, dedicated sites, Facebook pages, etc.). Only accounts including explicit statements pertaining to personal experiences as a victim of gang-stalking (e.g., “I am being gang-stalked”; “I am a targeted individual”) were considered eligible for inclusion in the study. Accounts were required to exceed a minimum length of 250 words to ensure that there was sufficiently detailed material to analyse [25]. Accounts were not included where there were indications that the writer was below the age of 18. Only material in the English language was included in the study. Two researchers examined each personal account, and cases were only included in the study where there was agreement between the two that they met the above-stated inclusion criteria. The first 50 cases to meet the inclusion criteria were adopted. Saturation of information was deemed to be met following analysis of 40 cases. This was indicated by no new categories emerging from the data. Where applicable, participants were de-identified by having their associated identifiable information removed from their self-published accounts prior to analysis.

### 2.2. Content Analysis

In order to gain insight into the subjective experiences of self-defined victims of gang-stalking, a content analysis was conducted [26]. Content analysis is a research method that provides a systematic and objective means to make valid inferences from verbal, visual, or written data in order to describe and quantify specific phenomena [27] (p. 314). It provides a systematic and objective means of making informed inferences from data via the use of categories, a category being a group of content that shares a commonality. Content analysis allows for a broad range of categories to be generated to reflect participant responses. It does not require that all data be coded, particularly when there is high inter-rater agreement on the categories generated [27].

No categories were established a priori. In order to generate the initial categories, two of the authors separately coded 10 gang-stalking accounts. Following this, we collaborated to generate a list of categories to which subsequent participant experiences would be allocated. This was an iterative process, with categories subject to refinement throughout the remaining analyses. Inter-rater agreement (absolute agreement) was at more than 95% across the categories generated, with consensus reached on disagreements via discussion. Validity was tested by checking the categories back against the original text to ensure that clear examples from each category could be identified in the original text.

Content analysis may encompass both a qualitative and a quantitative methodology [28]. Once the categories were established in the present work, they were expressed using both percentages and actual numbers. This allowed us to describe what the study population had written, and to describe what was both visible and obvious within the accounts [28]. Again, two of the authors performed these analyses separately. Inter-rater agreement (absolute agreement) was at 97% and, for the small number of disagreements, consensus was again reached via discussion. After the coding, some standard descriptive data were also gathered (length of stalking, reasons for gang-stalking).

### 2.3. Statistical Analysis

Comparisons of the categories generated through the content analysis in the current study were compared with data collected via a survey method [5]. Respondents to the Sheridan and James survey had provided qualitative responses to a series of questions about stalking victimisation. These responses were collated for each self-identified gang-stalking victim (*N* = 128) then checked against the categories created in the current study to produce values for their presence in each category. Two independent contributors undertook this task of establishing ratings, and inter-rater agreement was determined to be more than 95% across the categories generated. For the small number of disagreements, consensus was reached via discussion. The data from this study and those generated from the sample of Sheridan and James (2015) were entered into an SPSS database (version 25, IBM Corp., Armonk, NY, USA). The presence or absence of specific categories of subjective experiences were compared between the two samples, with chi-square analyses with a Yates correction being used to test for statistical significance and Cramér’s V used as an effect size measure.

## 3. Results

### 3.1. Length of Stalking

None of the writers of the narratives described their experiences as having ended. All 50 authors stated or implied that they had been gang stalked for lengthy periods of time (e.g., one mentioned being seen by seven psychologists during the period of being targeted, another described having been targeted whilst living in three different countries). The shortest case was described as having begun “in the last few months” and the longest as continuing for “more than 22 years”.

### 3.2. Reasons for the Gang-Stalking

The narratives were examined to establish whether the perceived reasons for being targeted by gang stalkers could be identified. Reasons were found in 20 of the 50 accounts. In all 20 cases, no named person was believed to be targeting the victim. Representative examples of the reasons given for the gang-stalking were as follows:

“Why? To drive people to meet-up groups or a psychiatrist so they can be medicated and/or get more federal grants for more mental health instead of training a real police force. Post Traumatic Stress Disorder is a big seller in the area.”

“It is a brainwashing military experiment. Post 9/11, they isolate certain people to stop them acting against government.”

“It is part of an overt agenda to create and test mind control. They are creating weaponry tested on us.”

“I began driving a friend around to make his pot sales and I started noticing we would get tailed from time to time. I knew this friend had connects in weird places (he knows members of Anonymous, he knows people who work at the Pentagon). I also did a lot of research and was very vocal about being anti-government and anti-corporation. This is what got me targeted. If you are vocal about your positions, then you will eventually run across a civilian spy who will pass the information to their superiors, who will continue to pass the information up the chain of command. Once you’re on their list, your life can become a living hell.”

“I am watched for 30 years after they put in the implants to see what the implants did to me.”

“Because I refused to join their devil cult and become an operative, I became a victim. My invitation to join came at an early stage via voice-to-skull.”

### 3.3. Categories of Experience of Gang-Stalking

Twenty-four categories of experience were identified in the 50 accounts of self-identified victims of gang-stalking. These are set out in Table 1, together with the proportions describing them. Given that the purpose of the study was to elucidate the phenomena constituting subjects’ experiences of being gang stalked, each category is illustrated in the table using descriptions from the case accounts. Spelling errors in the accounts have been corrected to aid comprehension. The most frequently experienced categories concerned being physically followed and/or surveilled (94%) or being the target of a conspiracy (80%). Two-thirds claimed that they were subject to “physical interference, intimidation, and harassment”.

The categories arrived at fall into six distinct groups. (The numbers refer to the items/examples in Table 1).

*i)*  Those that involve an invasive attack on the subject’s body
  Being remotely controlled/mind control (7); physical ailments as a direct result of gang stalking (10); voice-to-skull transmission (18); and control and surveillance devices implanted into body (20).
*ii)*  *Those involving an exterior attack on the person of the subject or their senses*
  Physical, interference, intimidation and harassment (3); targeted by noise (6); and physical attacks (21).
*iii)*  Physical interference with the individual’s personal environment or possessions
  Physical surveillance/being followed (1); electronic surveillance (5); subject to electronic hacking (8); subject to clandestine, unauthorised entry to home (13); vandalism/theft of property (14); and family and friends also targeted (17).
*iv)*  Assault on reputation
  Targeted by slander/gossip (11).
*v)*  Individuals or agencies involved in perpetrating or collaborating with the gang-stalking
  Police as part of the conspiracy (15); neighbours as part of the conspiracy (16); family/friends as part of the conspiracy (19); producing ‘evidence’ of gang-stalking fails to persuade authorities to intervene (22); and medical practitioners as part of the conspiracy (23).
*vi)*  Items concerning interpretation of the meaning of gang-stalking
  Victim of a conspiracy (by multiple agencies) (2); establishment cover-up (4); victimised as part of a global phenomenon (9); reinterpretation of past events in the light of the gang stalking experiences (12); and complained they didn’t know why they were being stalked (24).

### 3.4. Number of Categories Experienced

None of the authors of the narratives described experiences belonging to more than 20 of the 24 categories listed in Table 1. Of the authors, 14% described experiences from 15–20 categories (with 6% of the population describing experiences from within 20 categories), 27% described experiences from 11 to 15 categories, 30% described experiences from six to 10 of the categories, 26% said they experienced between two and five of the categories, and 4% of the authors of the narratives described experiences from within just one of the categories.

### 3.5. Sequelae of Gang-Stalking

The most commonly observed category of the reported effects of gang-stalking on individuals was psychological damage (42%), followed by isolation and loneliness (34%), and a determination to fight back (32%) (Table 2).

The categories fell into three groups (numbers refer to items/examples in Table 2).

*i)*  Psychological/physical effects
  Psychological damage (1); isolation and loneliness (2); resentment/distress at being treated as crazy or paranoid (4); physical ailments as a result of stress caused by gang-stalking (8); and feelings of hopelessness (11).
*ii)*  Practical effects/losses
  Changed lifestyle (6); financial losses (7); efforts to escape from gang-stalkers (10).
*iii)*  Fighting back
  Determination to fight back (3); found support from other gang-stalking victims through the internet (5); and development of hatred/violent tendencies (9).

### 3.6. Number of Sequelae Experienced

Only 14% of authors of the narratives did not mention any sequelae. The largest proportion (66%) scored positive for experiences of one to five categories. The remaining 20% endorsed nine or 10 categories. None endorsed all 11.

### 3.7. Comparison between This Study and Cases from Sheridan and James (2015)

#### Comparison of Phenomena

Table 3 compares the two samples, specifically the categories of experience produced through content analysis in the current study and the same categories applied to materials from the 128 cases in the Sheridan and James [5] questionnaire study. This comprised free text, ranging in length from 187 to 3712 words (median 571, mean 627). The categories of phenomena identified in the current study were found to have been experienced by cases in the earlier study. The tables show that, in addition, despite the differences in methodology between the two studies, there were no significant differences between the proportions of self-defined gang-stalking victims reporting phenomena in 16 (66%) of the 24 gang-stalking experience categories. Significant differences in eight items all concerned greater proportions of particular phenomena being experienced by cases from the current study sample. These comprised four forms of reported direct physical interference (physical interference, intimidation and harassment; vandalism/theft of personal property; physical attacks; and family and friends of victim also targeted) and four categories concerning interpretation of the wider meaning of events (victim of a conspiracy (by multiple agencies); establishment cover-up; victimised as part of a global phenomenon; reinterpretation of past events in the light of the gang-stalking experiences).

The numbers of categories experienced were calculated for both samples. Counts were merged into four groups (1–5, 6–10, 11–15, and 16–24 of the experience types). No significant difference was found between the two study samples in chi-square testing.

### 3.8. Comparison of Sequelae

The same categories of sequalae were found in cases from the earlier study [4] as in the current study (see Table 4). Comparing the frequency of the categories relating to sequelae between the two groups, there were no significant differences for six of the 11 categories (55%). A significantly greater proportion of cases from the current study sample described experiences from the following five categories: isolation and loneliness; determination to fight back; resentment/distress at being treated as crazy or paranoid; found support from other gang-stalking victims through the internet; and financial losses.

The numbers of categories of sequelae experienced were calculated for each sample and merged into three groups for analysis (0–3, 4–7, and 8–11 types of sequelae). There was no significant difference between the two study samples in chi-square testing.

## 4. Discussion

Given that the available evidence suggests that 12% of stalking reports involve gang-stalking and that, at a conservative estimate, 8% of women and 2% of men report being stalked at some point in their lives, it would appear that the subjective experience of being gang-stalked could affect around 0.66% of adult women and 0.17% of adult men in the western world at some point in their lives. It might be assumed that something that affects the lives of so many people would have been the subject of extensive research. However, this is not the case.

This is the first study to examine the phenomena of the gang-stalking experiences using a methodology that allows categories to emerge de novo from subjective descriptions. The only other empirical study of gang-stalking phenomena, of which we are aware, required subjects to fit their own experiences into categories derived from earlier studies of those stalked by individuals [5]. The categories of experience arrived at through the content analysis in the current study are therefore the clearest available expositions of the core phenomena of gang-stalking. The categories are unlikely to be exhaustive, given that they are based upon the phenomena that the individual subjects chose to report. However, they are likely to constitute those concepts that the individuals considered the most important. As well as extracting categories from the data, this study also grouped data into types, offering the first empirical attempt at a phenomenology of the gang-stalking experience.

Inevitably, this exercise is subject to the limitations of its methodology. The study concerned 50 descriptions of gang-stalking experiences taken from the internet. Yet, whilst not being an ideal source of data, this is one of the few sources available through which the gang-stalking phenomenon can be studied. Data from the internet is convenient and readily available, both to the current authors and others who might wish to replicate this study. Exploratory studies of this type are of value as there is currently little information available to guide agencies that encounter individuals presenting with these complaints. Such qualitative analyses of online forum content have been used previously in studies seeking to characterise perceptions and experiences within populations and topics that are not well understood [29,30]. The study of gang-stalking experiences must inevitably adopt an exploratory approach, given the lack of published studies in this area. As for the sample itself, the study cases were selected as the first 50 internet searches to satisfy the inclusion criteria, which was approximate to a random sample of such descriptions from the internet, although potential priority effects of the algorithm used within the search-engine cannot be excluded. It could be postulated, however, that the internet itself may act to shape experiences of gang-stalking phenomena, given that it is the principal source of information for sufferers and it may constitute a “closed ideology echo chamber” [31] where they find one another’s beliefs validated and reinforced. The possibility also exists that the results returned by the Google search may have been influenced by previous searches conducted on the computer in question. However, given the nature of the search (looking for descriptive accounts), the authors consider that this was more likely to have tightened the specificity of the search than to have introduced bias into its results.

The validity of the gang-stalking phenomena extracted from the current study sample is supported by the comparison with earlier results obtained from a self-selected sample using a questionnaire methodology [5]. Qualitative survey responses from Sheridan and James’ [24] study were classified as part of the current exercise into the categories produced by the present study and compared. Chi-square analysis showed more similarities than differences between the two samples, and all categories generated by the present study were endorsed by respondents to the Sheridan and James’ [5] questionnaire. In effect, the two studies produced descriptions of the same core themes.

Examination of the sequelae of being gang-stalked also produced similar categories to the study by Sheridan and James [5], supporting the conclusion that the two studies were examining similar phenomena. The earlier study comprised a more-detailed and substantial exploration of the effects of being gang-stalked, given that it used a battery of specific questions as well as a PTSD rating scale to explore experiences. Whereas this was a weaker methodology in terms of eliciting the phenomena that constitute the gang-stalking experience, it is a superior methodology when examining symptoms and consequences where the subject matter (in particular psychological state) is appropriately explored through standardised questions. It is clear from both studies that gang-stalking has a serious deleterious effect upon individuals’ well-being.

A factor that stands out in the current study concerns the aggression with which sufferers responded to their experiences (see Table 2). Of respondents, 32% described experiences falling into the category “determined to fight back”, while 16% made statements that fell into the category “development of hatred/violent tendencies”. Examples include: “We can either go out on our feet or our knees and I plan to sell myself for as high a price as I can reap from them”; “I may have to kill them before they kill me”; and “I think the only way forward is to get some weapons and act”. Violence in those who have complained of gang-stalking is not unknown. Sarteschi [21] described the cases of four men who believed that they were so-called “targeted individuals” and that the organised efforts of those who targeted them constituted “gang-stalking”. Collectively, the four men killed 28 people and injured 12 more. Sarteschi points to a need for intervention to prevent violent responses, and notes that the men whose cases were detailed in her study had engaged in significant efforts to make others aware of their perceived victimisation, these efforts taking the form of manifestos, videos and audio recordings, and social media posts. The violence perpetrated by these men was motivated by self-defence (in the form of a pre-emptive strike) and a need to alert the world at large to the dangers posed by gang-stalking. Serious violence appears to be rare in those experiencing gang-stalking phenomena. However, it is of note that the experiences grouped above under “invasive attack on the subject’s body” constitute examples of threat-control-override symptoms, which have been linked with an increased risk of violence [32,33]. It is also noted that the experiences and quotes above would satisfy criteria for the red-flag violence risk items of “homicidal ideation” and “high-risk phenomena” in the stalking and threat risk assessment guides the Stalking Risk Profile [34] and the Communications Threat Assessment Protocol [35].

## 5. Conclusions

The experience of being gang-stalked appears to be a widespread phenomenon that has been subject to little scientific examination. The current study provides a preliminary description of the phenomena involved that was produced by a methodology that did not incorporate pre-conceived assumptions. This provides a foundation upon which further research could be built. It also serves to confirm the harmful effects of the gang-stalking experience upon sufferers, first set out in the only other study available [5]. These findings constitute a potent reason why gang-stalking should be regarded as an important subject for study.

Whilst it was important to adopt a methodology that allowed the phenomena constituting the experience of gang-stalking to emerge de novo, it would now be appropriate to conduct studies of cases based upon specific questions in order to gain a clearer idea of the proportion of sufferers who experience each category of phenomenon, as the main categories have now been elucidated and the core phenomena described. This is because higher proportions are likely to be elicited through direct questioning than were found by studying internet descriptions. Finally, whilst this study has described the core phenomena of the gang-stalking experience, the question remains as to whether gang-stalking is a single phenomenon or represents several overlapping phenomena, each with its own defining pattern of experiences.

## Figures and Tables

**Table 1:** Content analysis of self-defined gang-stalking victims’ accounts according to their subjective experiences.

Category/Code: Frequency (Percentage) of Victim Responses. Exemplar Quotes.
*1. Physical surveillance/being followed: 47 (94%)*
“Some were following me when I went to restroom or cafe. Some people jumped on me while I was walking along corridors of the office building and pretended like accidents.” “I also would be followed to the library by people wearing disguises (elaborate, not just a wig and glasses)—sometimes dressed as other races even, makeup and all.” “Lately the following has gotten so extreme that cars tailgate with drivers openly speaking into walkie-talkies, and at supermarkets where individuals with heavy-duty black earpieces saunter up to my shopping carts and note what I am purchasing and walk away.” “Followed by on-the-job criminal investigation, followed on foot/car, by police officers.” “The perps who follow me around are trying to make me believe I’m being hunted/eaten by hybrid chimps and gorillas.” “As I drive down the road, multiple cars will start up and follow me.”
*2. Victim of a conspiracy (by multiple agencies): 40 (80%)*
“I believe that it is the Government that is behind it all with the help of the local police and other local agencies.” “People are spied on. Fusion Centers, Red Squads, police and sheriffs, people recruited by Homeland Security through Craigslist videos, people paid by the FBI stipends, people with clearances.” “It’s true, the stalking is non-stop, round the clock, world-wide, controlled and manipulated by police and every doctor, every shop, every store, every neighbor, while all the actor ‘friends’ turn away leaving me to be attacked. Most or all of the websites for targets appear false to me, with disinformation galore and trolls writing misleading articles and attacking people trying to communicate.” “Police cars, army trucks, navy and air force vehicles show up more often when I go out. When I was bullied at my previous company, some police cars followed me for no reason for a mile or two. However, I am not exactly sure who are at the top. They could be some powerful people. Or it could be rogue elements of intelligence agencies around the world.” “And there’s a wonderful feeling in achieving a result against an enemy who has everything on their side, from the police, councils, MI5, government and all those ‘normal’ people from all walks of life.” “Who funds this crap? Are you sitting down? Do you have an ability to see beyond the obvious? If not, you are wasting my valuable time. Here is the list: Insurance companies, Government agencies which include Law enforcement, Military, Disability companies, Workers compensation, Pharmaceutical companies, Very rich individuals that have a vested or non-vested interest in another individual. CIA, FBI, Homeland security, Terrorist cells. To name a few.”
*3. Physical interference, intimidation and harassment: 33 (66%)*
“Some of the harassment tactics are strangers blocking my way using grocery carts, invading my personal space, cutting me off while I was walking or driving.” “These last months, I have seen my stalkers become extremely aggressive. I have been forced off the road numerous times while driving my cars. I have had cars come straight at me, making it appear they are creating a head-on collision to scare me.” “I have been drugged, set up, harassed, mobbed, pestered, bumped into, bullied.” “I mean, at least 50 people are involved just watching me going to and from work, shining lights in my face!” “All the cars running into each other to harass me. The cars run into each other practically running over people to get to me. It’s so nice to be so popular!” “And every day now they are knocking into me in the street and in all the stores, bothering me and pushing me with their hands always.”
*4. Establishment cover-up: 32 (64%)*
“The authorities finance, protect and organise the stalkers, and the media blank any coverage of it.” “Don’t ever think of going to any authority because it is known at the highest levels and veiled.” “All the big organizations are part of it and they all cover it up. You name them, I will tell you. FBI, NRA, CIA. I just found out the Food Safety and Inspection Service are part of this.” “They have infiltrated all levels and that is why they can hide their actions... I need to be fast as they will wipe this more or less as I type, but some examples I will offer... All government departments, all media, airlines, US air force, the medical establishments, banks, large stores, companies who make weapons.” “I think that the Department of Justice is the main agency behind the cover up.”
*5. Electronic surveillance: 30 (60%)*
“The residence I live in was electronically bugged or I was put under audio and visual surveillance…” “Furthermore, to be stalked 24/7 by electronic devises, harass and invade my space and privacy rights. Further, to monitor by surveillance, use chemicals, energy to exploit me behind the scenes.” “I have been a victim of 24/7 covert electronic surveillance.” “That’s when the residence I live in was electronically bugged or I was put under audio and visual surveillance inside the so-called home I live in.” “Irradiating people with the satellite and over horizon radar grid which is through wall interferometry to scan/spy/assault/mind control, etc.”
*6. Targeted by noise: 22 (44%)*
“Sometimes it is hard to sleep because of noise harassment. Sometimes I actually use ear plugs when I go to bed.” “As I write this, the persecution is happening to me right now, via noise techniques: gang-stalkers living as wall-sharing neighbours, timed door-slamming, coughs, laughs, toilet flushing.” “I am studying in school and numerous noise campaigns happen” “The same sound is being heard since 20 years in different homes, different cities, and different states. The whole day this stone is used at different times in a characteristic manner. Sometimes every 5 minutes sometimes continuously, sometimes loudly.” “Using codified noise references during my orgasms as an inference of threat of physical torture.” “Noise and mimicking campaigns. Disrupting the target’s life, sleep with loud power tools, construction, stereos, doors slamming, etc. Talking in public about private things in the target’s life. Mimicking actions of the target. Basically, letting the target know that they are in the target’s life. Daily interferences, nothing that would be too overt to the untrained eye, but psychologically degrading and damaging to the target over time. In my case, I have not noticed this since London is quite noisy anyway and I know whom to contact after years of working for local authorities the moment a perp attempts this tactic. For a time, pebbles were thrown over the roof of my house in the middle of the night until I installed CCTV cameras.”
*7. Being remotely controlled/mind control: 20 (40%)*
“People have no idea of just how far advanced the authorities are in mind control methods and the means they have to apply it.” “I have been a victim of 24/7 covert electronic surveillance, psychological harassment, manipulation and mind control.” “They are fully able to take control of everyone’s thought processes. They did it in Nazi Germany and it is happening here right now.” “They cover you in chemtrails and you can’t scrub them off even using vitamin scrubs and these then allow them to take control of your mind and your thoughts.” “The mind control tactics are highly sophisticated, and they have Orders that make you see reflections of yourself.” “They insert thoughts and ideas and it took me a while to work out that these were not my thoughts, not my own ideas.”
*8. Subject to electronic hacking: 19 (38%)*
“All my passwords, emails, logins basically all my private info is compromised.” “The perps hack into not only my telephones but all the cell phones of my family members too in order to obtain information about my travel plans so that they can arrange people at strategic locations to demonstrate street theater in which well-rehearsed live dramas takes place.” “My computer has been hijacked, mobile and LAN phone.” “My ex-husband’s and my cell phones were cloned.” “The gang-stalkers have followed me to a friend’s house and hacked my cell phone to listen to the conversation inside and put my mother’s number on it as if she’d called. She hadn’t.” “After HOURS of researching again, tonight I somehow found myself here. I had to Register to post this comment, but before I got my email to confirm, they had already changed my password. They are That Quick and That Proficient.”
*9. Victimised as part of a global phenomenon: 19 (38%)*
“Also, I think this is a collaborative work of intelligence agencies around the world.” “This global program is real and just because it’s not happening to them doesn’t mean that it’s not happening at all.” “The government know and this is not a theory, there’s evidence globally past and present.” “They run the world, all the people in power are them...” “I have many instances, and so do thousands of others around the world.”
*10. Physical ailments as a direct result of gang-stalking: 18 (36%)*
“Skin is peeling off, my scalp has been burned quite a few times. I have been poisoned, diarrhoea for seven days straight until I was able to get medicine.” “There were many nights that I was gassed and electrocuted and I did not think I would live long enough to see the daylight hours the next morning.” “So much gamma rays and irradiation pumped through me and it hurts.” “I have undergone extreme poisoning within my home. My body literally has been shocked more times than I can count. The most occurrences happen when I try to go to sleep at night. Sleep deprivation is, I am assuming, one of their favourite punishments. My skull has been fried and replaced with who knows what. At one time, I could barely leave my house because of the ‘melting’ feeling in my head. They constantly play with my heart as if it is a bean bag and it has also been subjected to their very joyful shock treatment. Sometimes I cannot even feel a heartbeat. The feeling of it being removed and replaced was the eeriest feeling of my life. Once they removed it, I could not feel any part of my body and the ceiling darkened as if I were really dying until they decidedly plugged it back in. I have undergone consistent harassment. I have also undergone constant pressure to my head as if it is being squeezed. Even while driving.” “They hijacked my neurons and it made them ache: you can’t have poison and electronic interference at these levels without physical trauma as consequence.” “I haven’t been able to breathe outdoors for years. That’s how it all started. I woke up drowning in mucus every night for 4 1/2 years... When I go outside, even for only a minute, the chemtrail toxins STICK to me like a magnet and the smell is so strong I have to scrub the hell out of my skin to get it off.”
*11. Targeted by slander/gossip: 17 (34%)*
“This is done via slander campaigns and lies. E.g., people in the target’s community are told that the target is a thief, into drugs, a prostitute, paedophile, crazy, in trouble for something, needs to be watched. False files will even be produced on the target, shown to neighbours, family, storekeepers. Attempts of this had been made against me with limited success. Often people who know me or individuals at places I frequent have given me CCTV footage or descriptions of the perpetrators who try to slander me.” “Slowly they were able to turn many people against me by spreading all sorts of lies and rumors.” “More and more people are trying to cause accidents around me, people at work almost seem bi-polar—one minute they are friendly, the next they are telling lies about me to many people.” “If any man starts to go on about the slander about you (because there always is), just walk away.” “It is the non-stop constant lies all about my life and they are telling to everyone I am seeing.”
*12. Reinterpretation of past events in the light of the gang-stalking experiences: 17 (34%)*
“I remember those strange occurrences…they didn’t give me a sense years ago, but now I’m able to fix the mosaic.” “Now, I think back and I think he may have just been saying it so he could carry on with what he was doing.” “I left the city because of the suffocation problems, because I thought it was just pollution, but now I know better.” “I had been bullied at many different places. I thought they all happened because of my fault or I was just unlucky. Now I know that my perpetrators have been denying my basic human rights for years. They have been sabotaging all my opportunities. They have been ruining my personal/social/dating relationships. They have been doing lots of evil acts. They have been trying to push me into the lowest level of the social ladder.” “All along I had doubted myself and then I had a revaluation (series of revelations/re-evaluations) that enabled me to see they had been running things this way for me.”
*13. Subject to clandestine, unauthorised entry to home: 17 (34%)*
“I’ve had people coming into my house when I’m not home. Even when I’m home. Take nothing. Move a couple things around is all…my attic space always looked different each time I’d go up into it.” “At this time, I started noticing strange things happening INSIDE the residence I was living in, my HOME. Personal things started showing signs of having been gone through by someone. My books, personal papers, journals etc..., had pages folded, were marked in various places or in some cases were actually missing.” “Now I can’t even leave my house because as soon as I am home they are all talking, and then they are inside going through all my personal papers and moving my undergarments.” “And they will enter your premises and move things and alter things to just let you know they have been there.” “Every time I went to the store, the network was inside my bathroom. They have this crazy need to check my cabinets and I can hear them whispering ‘she is coming home, let’s leave’.”
*14. Vandalism/theft of personal property: 16 (32%)*
“Wires of my car were badly damaged while it was at a garage overnight.” “One of the first incidences was finding items ruined that I couldn’t explain.” “My vehicle has been broken into and vandalized.” “They are all about causing property damage and bugging cars.” “My car broken into. The locks to my house door punched out. My screen windows cut in order to gain access. The list goes on and on.” “Breaking up my dog kennel was one of the final straws.”
*15. Police as part of the conspiracy: 16 (32%)*
“The police turn a blind eye.” “A state police helicopter would hover over the library and then follow me home.” “Even when this was done directly in front of police, in their plain sight, they, too, upon my approaching them would pretend like they did not see it.” “I believe that it is the Government that is behind it all with the help of the local police and other local agencies.” “The truth is that illegal surveillance and illegal police stings are taking place. Plus let’s not forget about government watch lists that are being sent to your local law enforcement. The lists can also be a local law enforcement list.” “And now the local police department has gotten involved. They are constantly following me. Not pulling me over and ticketing me for anything, just following me just about every day.”
*16. Neighbours as part of the conspiracy: 13 (26%)*
“The electronic harassment and everything else comes from your neighbours.” “Since 2012 I began to find out some of the people, neighbours I’ve known all my life were involved.” “My neighbors started to harass me in various ways. One on right above my apartment started jumping on the floor at night. Another one banged my wall while I was sleeping. Some neighbors frequently slammed the doors.” “As I have stated—some of my neighbours are gang stalking me.” “I ended up in tears this morning after another argument with perp neighbours!” “But certainly, many of my neighbours (not all) are involved, now.”
*17. Family and friends of victim also targeted: 13 (26%)*
“Far too many of my own friends are being slandered and attacked in this manner to ignore this now.” “My best friend has now suffered threats, harassment at work and through his landlord. He has had cars driving around him, the same way they do me. He has had strangers outside his house and look into his flat (with me as witness). Some neighbours now hate him. Everything that has happened to me, only in a less intense way.” “My family and friends have been harassed and stalked as well, also extended family. Their cars have been tampered with. There have been third party attempts to monitor their credit card spending.” “Then, during the night they entered the home and left objects for me to find to let me know my friend was in danger.” “Recently when a relative died, I got three blocked calls that day which leads me to believe they are cyberstalking my family.” “My dad has been a target for decades. My grandpa (dad’s dad) was a freemason. Dad said grandpa got shot in the chest with some sort of weapon and died from a heart attack.”
*18. Voice to skull, or V2K, transmission. A widespread belief among targeted individuals that mind control technologies, based on microwave signals, are used to transmit sounds and thoughts directly into their heads: 13 (26%)*
“They automate the V2K. It is repetitive and constant. It is comprised entirely of insults and instructions to “Go”, “Get out”, “You better get out of here”, “Dead”, “You’re in trouble” and “Out”. Over and over and over.” “v2k is speaking from a man on the business end of it for four years now the most insidious evil technology ever concocted.” “I’ve also been brainwashed via v2k in the past, to believe, alien species, i.e., insectorpeeds, anthrapods and huge arachnids are going to eat me, it gets scary at times because if you hear something for so long it starts to affect you.” “I ‘hear’ the voices of people who interrogate me and maltreat and torture me around the clock.” “See I become aware of this only the past 12 years since they started to give me voice to skull and gang-stalk me.”
*19. Family/friends as part of the conspiracy: 12 (24%)*
“All my family is in on it, as your own family will be. You can’t trust anyone.” “My family participates in this horrendous low life crime against all decent men and women.” “My fiancé of 8 years was turned and now I am alone.” “Next thing you know, family members and friends are cutting all ties with me. They do not want to speak, they will not tell me why, and they will not explain what their decision is based upon.” “My only Weapon is going forward and expose them (Swiss Government/Town of Zürich/Churches of Zürich, my own Family and their Friends.”
*20. Control and surveillance devices implanted into body: 12 (24%)*
“They inject Nano microchips in the vagina and make circuit boards there with them.” “I consider myself lucky in one way, if I can say that, I was surgically mutilated more than 30 years ago—and I think that since than the implants in me loosened up a little bit—I went to sauna, went to baro-chamber and did many crazy things in desperation.” “I am curious about how a person like myself might get into contact with someone who can help to remove implanted devices, evaluate for implanted devices and also to network with others to take a proactive approach to halt this sort of thing.” “I damaged the nano-cameras in my eyes by stretching the eyelid. I did the right thing. Be careful not to damage your eyes if you do the same.” “They are building cyborg-techniques inside of me. I don’t know how, but they are doing it wireless.”
*21. Physical attacks: 11 (22%)*
“I have been physically/sexually attacked by four men over the course of my gang stalking.” “I have undergone extreme poisoning within my home. My body literally has been shocked more times than I can count. The most occurrences happen when I try to go to sleep at night. Sleep deprivation is, I am assuming, one of their favorite punishments. My skull has been fried and replaced with who knows what.” “My scalp has been burned quite a few times. I have been poisoned, diarrhoea for seven days straight until I was able to get medicine.” “There were many nights that I was gassed and electrocuted and I did not think I would live long enough to see the daylight hours the next morning.” “Oh yes, they beat me with hard sticks. I have the bruises, I am in pain.”
*22. Producing ‘evidence’ of gang-stalking fails to persuade authorities to intervene: 10 (20%)*
“I have 7 psychologists who already evaluated me confirming I was gang stalked.” “However, I have spoken to police officers who freely admit they know it’s happening and in their area. This proves it’s a very real experience, not down to medication in any way.” “I have tons of pics of them doing it.” “No matter how much evidence I produced, it would be ignored and the attacks would be increased.” “Photographs and car registration numbers amongst other privately collected evidence has been submitted to my local police CID office.” “Thousands of pieces of evidential material are in my possession. But for what good? They are all part of it, so I can’t show anyone.”
*23. Medical practitioners as part of conspiracy: 10 (20%)*
“So, over a 2-year period I was taken from my home in hand cuffs 4 times and finally diagnosed as, you guessed it, paranoid schizophrenic.” “To be labelled as delusional has ruined me. The psychiatric cabal got what they wanted.” “I was sick of hearing voices. I thought I might be able to report it and get somewhere back to a normal life ... WRONG... they put me into a mental ward and I stayed for 3 weeks.” “And I was the one put into the institution and that is sick, sick.” “Then they diagnosed me as mentally ill for their profit.”
*24. Complained that they didn’t know why they were being stalked: 8 (16%)*
“I clearly don’t know why they targeted me at the first place.” “But why would you make a single woman with two children suffer to that extreme, especially if you have had no personal relationship with them. Can we say personal vendetta to the maximus. Grudges should never run that deep.” “I just want to know WHY.” “I always wondered, why did they hate me so much? …sometimes I cried because of it…” “I have no idea what started it all and why.”

MI5—The United Kingdom’s domestic counter-intelligence and security agency; CIA—Central Intelligence Agency, USA; FBI—Federal Bureau of Investigation, USA; NRA—National Rifle Association, USA; CCTV—Closed circuit television; LAN—Local area computer network.

**Table 2:** Content analysis of self-defined gang stalking victims’ accounts of the sequelae of their experiences.

Category/Code, Frequency (Percentage) of Victim Responses Exemplar Quotes
*1. Psychological damage: 21 (42%)*
“I hate my life with such a passion that I am yet again depressed and sometimes the idea of death sounds inviting.” “From fear and confusion, self-doubt, anger, disbelief, shock, doubting my sanity, gloom, depression, isolation, bewilderment, and more.” “This issue, has brought a severe case of depression, that is starting to take a toll on my health, and my sleeping pattern is completely abnormal.” “I was stressed, drained emotionally, mentally and took off of work on a regular basis.” “Eventually I would crack and begin a death spiral into substance and alcohol abuse.”
*2. Isolation and loneliness: 17 (34%)*
“The biggest and most hurtful thing with gang stalking, is loneliness.” “I am lonely, friendless, and have no woman. I don’t even have other TI friends.” “I choose to isolate and I trust virtually nobody. I don’t even attempt relationships anymore because they all turn out to be perps.” “They have made it so I am totally alone.” “I had so many friends and now I am a hermit.”
*3. Determination to fight back: 16 (32%)*
“I’ve decided I can surrender, or I can fight, so I’m ready and prepared to have as big a mouth about this as I can.” “Well, after two years of work-place harassment and 1 year of being stalked by the police, I decided to fight back.” “So, they are losers from the outset, and will also be the losers if the theme “Targeted Individual” enters the public spotlight. That must be our goal as victims—these people and the issue to drag into the light, like it happened with Snowden.” “Please do let’s organize effectively. We can either go out on our feet or our knees and I plan to sell myself for as high a price as I can reap from them.” “Light up the darkness fight for the freedom that is our birth right and challenge the evil. We will not be broken.” “I don’t have much problem with the gang stalking anymore because I shot two of them with my wrist rocket and they are pretty cowardly anyway—won’t even come out and fight me in a secluded parking lot at night, though I’ve called them out dozens of times.”
*4. Resentment/distress at being treated as crazy or paranoid: 14 (28%)*
“Dismissing what I have lived with as nonsense is quite frankly offensive.” “Of course, no one would listen, and I would simply be ignored as being ‘paranoid’ or ‘crazy’.” “And let’s face it….. gang stalking is hardly in public consciousness as yet, so they will have the perfect cover to deny it! Hence the ‘you must be crazy’ epithets some of ‘They want you to sound crazy and be treated badly’. “ “…telling me that I am crazy and I need help.” “Every person I know believes I am mad”.
*5. Found support from other gang-stalking victims through the Internet: 13 (26%)*
“So, my only solace is visiting a few sites, this being one of them.” “First a thank you so very much for the presence of this website and community, the alleviation of stress, duress and despair by realising I am not alone has been of massive help, these past days.” “I remember I was desperately seeking some answers to my stalking experience. I was searching the net and then not getting any answers. I was searching everywhere. I was disturbed, alone and persecuted. It was perhaps in April 2008, I came across somebody having similar experiences to mine. I selected this site because of her.” “I suggest you go to the gang stalking website and read how you can help yourself.... NOW.” “I made my own support network and I talk with people around Australia and the world.”
*6. Changed lifestyle: 13 (26%)*
“I have changed all my habits including going to the supermarket, since the stalkers have gone to all public places and tried to intimidate me.” “No way can I live my normal life now. So many things I cannot do anymore.” “I can no longer attend church or go see a movie or any of the normal things that non TIs can do.” “My life has completely changed and harassment continues ever since.” “It hurts to admit this, but my life is now so different as a result of gangstalkers and I no longer go out.”
*7. Financial losses: 12 (24%)*
“These people will not leave and are there to help you reach financial ruin!” “The actual cost of time, money and damage to my good name.” “I have lost untold money amounts.” “Financial burden caused by bureaucrats that injured me emotionally, mentally and financially.” “They have enormous untold financial means and now I am in the position where I have no money because of it.”
8. *Physical ailments as a result of stress caused by gang-stalking: 10 (20%)*
“When all these things happened, I got sick couples of times. I had back pain, lose motion, headaches, breathing problems, allergies, cough. I could not sleep well.” “It was negatively impacting me mentally, emotionally, psychically, spiritually and physically. I was getting physically sick at times.” “I get a lot of chest pain from the worry of it all.” “The stress of it all is so bad that I can’t go to work and suffer with nausea and headaches pretty much full time.” “The anger and the stress and all the other negative emotions make me physically disabled.”
*9. Development of hatred/violent tendencies: 8 (16%)*
“I am to the point where I absolutely hate everyone and wish the worst death on all who gang-stalk me.” “I may have to kill them before they kill me.” “Sometimes I even imagine my stalkers being strongly tied, their mouths filled with socks and me beating the crap out of them by the heavy iron stick…I fantasize about killing them in many possible ways…and I’m a Christian…even though I know it is a sin to hate and kill, I can’t help these feelings…they simply come sometimes…very spontaneously…and I was never like that before….gang-stalking made me very aggressive…” “I am so full of hate now that I feel like I am going to combust.” “It makes me violent sometimes.” “I think the only way forward is to get some weapons and act.”
*10. Efforts to escape from gang-stalkers: 7 (14%)*
“English is my second language. I ran away to Israel, but the criminal FBI followed me.” “How many times have I moved? Three. Interstate. There is hiatus, but they resurface.” “I have lost everything trying to get away from them, but their reach is endless and they always find me.” “I have given up trying to get away from them now. You cannot hide. So, don’t even waste time running.” “For a long time, I made efforts big, big efforts to leave them behind. I travelled so far.”
*11. Feelings of hopelessness: 7 (14%)*
“I pray that my future will be different, but I have little hope that it will be.” “Learned helplessness has been their primary ongoing victory over me over these two years.” “Simply I feel like my life has no future at all….” “I really don’t think I have that much strength to put up with this that much longer.” “Every day I am more despairing, and they have broken me down.”

**Table 3:** Comparisons between content analyses of internet and questionnaire samples on phenomena constituting the experiences of being gang-stalked.

Category	Frequency and % This Study Sample (*N* = 50)	Frequency and % Calculated for Sheridan and James [5] Sample (*N* = 128)	χ^2^, *p*, Cramér’s V
1. Physical surveillance/being followed	47 (94%)	109 (85.2%)	ns
2. Victim of a conspiracy (by multiple agencies)	40 (80%)	41 (32%)	31.46, <0.01, 0.43
3. Physical interference, intimidation and harassment	33 (66%)	46 (35.9%)	11.96, <0.01., 0.27
4. Establishment cover-up	32 (64%)	23 (17.9%)	33.56, <0.01, 0.45
5. Electronic surveillance	30 (60%)	71 (55.5%)	ns
6. Targeted by noise	22 (44%)	56 (43.8%)	ns
7. Being remotely controlled/mind control	20 (40%)	51 (39.8%)	ns
8. Subject to electronic hacking	19 (38%)	35 (27.3%)	ns
9. Victimised as part of a global phenomenon	19 (38%)	8 (6.3%)	25.75, <0.01., 0.40
10. Physical ailments as a direct result of gang-stalking	18 (36%)	30 (23.4%)	ns
11. Targeted by slander/gossip	17 (34%)	26 (20.5%)	ns
12. Reinterpretation of past events in the light of the gang-stalking experiences	17 (34%)	21 (16.4%)	5.62, <0.05, 0.18
13. Subject to clandestine, unauthorised entry to home	17 (34%)	27 (21.1%)	ns
14. Vandalism/theft of personal property	16 (32%)	14 (10.9%)	9.93, <0.05, 0.25
15. Police as part of the conspiracy	16 (32%)	23 (17.9%)	ns
16. Neighbours as part of the conspiracy	13 (26%)	23 (17.9%)	ns
17. Family and friends of victim also targeted	13 (26%)	15 (11.5%)	4.51, <0.05, 0.18
18. Voice to skull, or V2K, transmission. A widespread belief among targeted individuals that mind control technologies, based on microwave signals, are used to transmit sounds and thoughts directly into their heads	13 (26%)	28 (21.8%)	ns
19. Family/friends as part of the conspiracy	12 (24%)	15 (11.5%)	ns
20. Control and surveillance devices implanted into body	12 (24%)	21 (24.2%)	ns
21. Physical attacks	11 (22%)	10 (7.8%)	5.66, <0.05, 0.20
22. Producing ‘evidence’ of gang-stalking fails to persuade authorities to intervene	10 (20%)	11 (8.6%)	ns
23. Medical practitioners as part of conspiracy	10 (20%)	11 (8.6%)	ns
24. Complained that they didn’t know why they were being stalked	8 (16%)	17 (13.3%)	ns

**Table 4:** Comparisons between content analyses of internet and questionnaire data on sequelae of being gang-stalked.

Category	Frequency and % This Sample	Frequency and % Sheridan and James [5] Sample	χ^2^, *p*, Cramér’s V
1. Psychological damage	21 (42%)	57 (44.5%)	ns
2. Isolation and loneliness	17 (34%)	10 (7.8%)	17.2, p < 0.01, 0.33
3. Determination to fight back	16 (32%)	10 (7.8%)	14.9, p < 0.01, 31
4. Resentment/distress at being treated as crazy or paranoid	14 (28%)	17 (13.3%)	4.4, <0.05, 0.18
5. Found support from other gang-stalking victims through the Internet	13 (26%)	6 (4.7%)	14.9, <.01, 0.31
6. Changed lifestyle	13 (26%)	27 (21.1%)	ns
7. Financial losses	12 (24%)	14 (10.9%)	3.9, <.05, 0.17
8. Physical ailments as a result of stress caused by gang-stalking	10 (20%)	23 (17.9%)	ns
9. Development of hatred/violent tendencies	8 (16%)	9 (7%)	ns
10. Escaping from gang-stalkers	7 (14%)	7 (5.5%)	ns
11. Feelings of hopelessness	7 (14%)	23 (17.9%)	ns
